# Routes to Lenition: An Acoustic Study

**DOI:** 10.1371/journal.pone.0009828

**Published:** 2010-03-23

**Authors:** Eftychia Eftychiou

**Affiliations:** Phonetics Laboratory, University of Cambridge, Cambridge, United Kingdom; University of Barcelona, Spain

## Abstract

**Background:**

Vowel lenition and its link with coarticulation have been the subject of extensive debate in the literature. The aims of the present paper are to demonstrate how vowel lenition and coarticulation are linked in Cypriot Greek (henceforth CG), to determine the nature of vowel lenition, and to illustrate how vowel lenition and coarticulation result from aerodynamic phenomena.

**Methodology/Principal Findings:**

Eight speakers were recorded producing utterances ending in either /i/ or /u/. Acoustic measures such as V_1_F2 and stop duration were employed to determine whether lenition of the vowels results in coarticulation with the preceding consonant. Results show that there is extensive stop-vowel coarticulation in CG and that stop production is as variable as vowel production, with full vowels never co-occurring with canonical consonants, indicating the existence of two routes to lenition in CG.

**Conclusions/Significance:**

These findings suggest that lenition in the final syllable is a consequence of the supralaryngeal articulation coupled with a marginal glottal setting.

## Introduction

The Greek language has five vowels underlyingly: /i ε 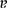 o u/. Northern and western varieties of Greek are well-known for the fact that the two close vowels tend to be elided when they occur in unstressed position in connected speech. However, experimental evidence suggests that close vowel lenition is also common in Standard Greek (henceforth SG) [1]–[6]. Differences in vowel production are not limited to Greek but are also common in a range of phonetically better-known languages, such as Brazilian Portuguese [7], European French [8], Japanese [9], [10], and even English [11].

Various terms have been employed to describe the non-modal ways in which vowels are realised in the languages of the world; *reduction* [4], *devoicing* [9], [10], [12], [13], *elision* [4], [11], or *devocalisation* [14]. This diversity in the terminology employed to describe vowel realisation is, perhaps, a reflection not only of the different ways in which a vowel is realised, but also of the continuum of changes that it goes through, from extreme shortening, to devoicing and, finally, to elision [4], [5]. According to Gordon [15], vowel devoicing operates on a continuum with token to token variation; on one end of the spectrum is a devoiced vowel and on the other end is vowel deletion, with various degrees of voicing falling in between. It will be shown later that *lenited* vowels, such as vowels without a complete formant structure, can exist in the language under investigation but, as some of these vowels are voiced, the term *devoicing* would not be appropriate in this case; *elision* would also be an inaccurate term as it refers only to the final stage of non-modal vowel realisations. *Reduction* is not preferred because of its relation to centralisation. As Payne points out, as a vowel becomes shorter, less peripheral or more devoiced, it also becomes more lenis [16]. For the purposes of this paper, the term used will be *lenition*, on the basis of the fact that this term is viewed as encompassing all possible realisations of the vowel. If lenition is considered to be a departure from the target [17], a vowel which does not exhibit all vowel-like characteristics can be considered as lenited.

### Vowel Realisation Continuum

Experimental studies of vowel lenition suggest that differences between modal and non-modal vowels can be found in their acoustic properties, such as their intensity, voicing, and spectral characteristics [15]. Lenition is most common in close vowels because of their intrinsic characteristics [9]. Close vowels are short in duration [18], and they create back pressure leading to a the reduced transglottal pressure drop which results in devoicing [19]. There has to be a minimum level of pressure difference across the glottis for voicing to exist, but sustaining voicing is difficult for close vowels because of the build-up of supraglottal pressure caused by the close constriction of the vocal tract for such vowels [20].

In Japanese, lenited vowels can be fricativised, in the sense of becoming vowel-coloured fricatives, with the frication produced by airflow through a narrowed oral tract providing the energy for vocal resonance [12], [21]. Research on a similar phenomenon in SG shows that vowels are manifested in different ways in connected speech, and go through stages such as extreme shortening and devoicing before they reach elision. The results suggest that even in the most extreme stages of the vowel realisation continuum, there are traces of the vowel retained in the preceding consonant, in the form of formant transitions, that inform the listeners as to the identity of the vowel [4] (see note 1 in Supporting Information File S1). These studies reveal that *vowel lenition* covers a spectrum of vowel realisations and that even languages considered to involve devoiced or deleted vowels actually involve vowels with a wide range of manifestations. Moreover, it appears that vowel lenition does not merely involve cases of vowels that have no appreciable voicing, but also cases of vowels whose formant structure survives even though the voicing is not realised.

### Coarticulation

The effects lenited vowels have on neighbouring consonants have also been investigated. Studies have examined the coarticulation of consonants and vowels and have, thus, sought to explain how it is that listeners perceive the missing vowels and how it can be established that these vowels might be masked by neighbouring segments without being deleted. Coarticulation is the superposition of multiple influences, stemming from acoustic-phonetic requirements of context and from physical interactions with other articulators, on the movement of an articulator [22], and is the cause of the variability and unsegmentability of speech [23].

There are two directions in which coarticulation operates. The first is anticipatory coarticulation, which has been hypothesised to reflect programming strategies for upcoming context [24], [25]. What this means is that anticipatory coarticulation is considered to be the outcome of a planning process that takes place at a more cognitive level in order to deal with the articulatory requirements of following sounds. The second direction in which coarticulation operates is carryover, which has been attributed to the mechanical/inertial properties of the articulators [24], [25]. In other words, carryover effects are caused by the conflicting requirements imposed on the same articulators. The primary concern of the present study is anticipatory coarticulation.

Consonant-vowel coarticulation has been studied extensively via a variety of experimental methods. Results show that, in VCV sequences, F2 transitions into and out of a consonant depend on the entire vowel context and not just on the consonants. Stop loci are not unique because the production of the consonant involves concomitant articulatory adjustments partially anticipating the configuration of the following vowel [26]. Apart from what has been found for stops, experiments on the coarticulation of fricatives with close vowels found that fricative F2 is higher when the fricative precedes /i/ than when it precedes /u/ [27], [28] and that centroid values vary as a function of the upcoming vowel context [29]. In sum, fricative spectral properties have been shown to be affected by those of adjacent vowels (e.g.[30]). This fricative-vowel coarticulation has been demonstrated to be perceptually salient as listeners are able to extract vowel information when presented with portions of the fricative [28], [31]–[33].

The perceptual salience of stop-vowel coarticulation has also been subject to investigation. In a series of experiments, Blumstein and Stevens [34] showed that listeners can extract both C and V information from stimuli that can be as short as one glottal pulse, as long as the burst is present, and that identification improves as the stimulus becomes longer. They further showed that listeners base judgments of vowel quality on the terminal values of the formant movements which, in the case of /u/ and /i/, approach the appropriate vowels (cf. [30]). Similar results have been obtained by Winitz *et al*. [35], Bonneau [36] and Repp and Lin [37].

### The Link between Vowel Lenition and Coarticulation

The link between vowel lenition and coarticulation stems from the fact that many listeners report hearing the vowels even though there is no visual evidence for them in the acoustic record, suggesting that traces of the vowel can be found in the neighbouring segments which cue the listeners as to the identity of the vowel. Both Dauer [4], [5] and Varden [21] report cases of the formant structure of a vowel superimposed on the frication of the preceding consonant. However, they also report cases of vowels for which there is no visual evidence in the acoustic record.

There are various studies investigating the influence of acoustic properties of elided vowels on neighbouring segments. Most base their argument regarding the continuing underlying presence of the vowel on durational data, by showing that the duration of a syllable which includes an elided vowel is longer than an identical one which does not (e.g. [11], [13]). These data indicate that the vowel is present in the adjacent consonants. Perceptual evidence for this claim has also been offered with listeners able to differentiate tokens solely on the basis of the duration of the consonant in cases where the vowel is not present in the acoustic record [38], and also able to differentiate words ending in different vowels even though the vowels are not visually present [12].

### The Nature of Vowel Lenition

An ongoing debate exists in the literature on vowel lenition concerning the question whether this phenomenon is phonological or phonetic. If vowel lenition is phonological, then the change between a modal and non-modal vowel must be allophonic. Therefore, allophonic variation is any change in the canonical form that constitutes the plan of an utterance. The difference between this and other processes that result in allophones is that the allophones created by vowel lenition are free variants and do not need to occur in separate phonological environments or be the result of a phonological rule [12]. If, on the other hand, vowel lenition is a phonetic process, then it is expected to result in gradient outputs, meaning that the vowel would be expected to go through many stages before visually disappearing from the acoustic record, as has been shown in previous studies [4], [5], [15].

Though traditionally such vowels were considered to be deleted, i.e. the process was deemed to be phonological whereby the vowel lost the feature [+voice] or [+syllabic], experimental research has revealed that the phenomenon would be more accurately described as being phonetic as there is evidence for vocalic influences on the consonant [11], [13], [21], [38]. Based on this evidence for vowel presence even when the vowel is not visually present in the acoustic signal, arguments have been put forth suggesting that the vowel is not gone but, rather, that its gesture is masked by overlapping gestures from adjacent segments [14]. Thus, most explanations concerning the nature of vowel lenition have been proposed within the framework of Articulatory Phonology (henceforth AP) [39]–[41], one of the main arguments of which is that gestures might be masked by other gestures but are never fully deleted.

Of course, the possibility that the phenomenon might be both phonological and phonetic should not be overlooked. Aoyagi [9] provided an overview of studies on Japanese vowel lenition, based on a variety of experimental methods, which provide evidence for both the phonological and the phonetic account.

### The Present Study

Arvaniti [42] has suggested that vowel lenition exists to a lesser extent in CG, but no other studies have looked at the phenomenon via experimental methods. Trudgill [43] has made a similar claim in stating that *high vowel loss* has not reached the Southern parts of the Greek-speaking world. The statement is true in that CG lenition does not form part of the process that occurs in Northern or Western varieties of Greek whereby mid vowels are raised and close vowels are ‘lost’, but perhaps not true because the process might occur to varying degrees in various parts of the South due to aerodynamic or other factors. In fact, impressionistic evidence suggests that the process is very prevalent in CG as non-native listeners report, for instance, that they perceive disyllables exhibiting this phenomenon as monosyllables (e.g. /tosi/ heard as [tos] (*this much, fem.*)).

As vowel lenition is hypothesised to occur in CG, it would be interesting to ascertain its behaviour, in order to determine where CG fits with respect to the cross-linguistic literature on vowel lenition and coarticulation. Firstly, it is important to determine whether the claim concerning the rarity of the process in CG holds by examining data from multiple speakers. Furthermore, an investigation of CG vowel lenition would reveal whether it behaves like vowel lenition in other languages in terms of resulting in a continuum of realisations before reaching the final stage of elision. Moreover, an examination of the link between vowel lenition and coarticulation in CG might reveal the vowels are not elided. Research into the link between vowel lenition and coarticulation in CG might also facilitate drawing parallels between CG and other linguistic systems which exhibit similar processes.

Finally, exploring vowel lenition in CG in the context of coarticulation might offer insight into the nature of the process in CG. Any possible influences of lenited vowels on neighbouring consonants would favour a phonetic description, most probably within the gestural framework drawing additionally from aerodynamic facts, whereas the complete elision of vowels whose presence is undetected in adjacent consonants would support the phonological interpretation.

The present experiment is motivated by the lack of phonetic studies on CG vowel lenition, as well as the lack of studies on Greek coarticulation in general. The acoustic experiment described in the following section aims to test the following hypotheses:

- Despite the claim that vowel lenition in CG is not very common ([42] for CG; [43] for Southern varieties), the present study predicts that the process is quite prevalent in the variety under investigation in light of the fact that non-native listeners claim to not hear certain vowels in CG speech.
- Native listeners, on the other hand, claim to perceive the vowel in question. On the basis of native speaker intuition, and in light of various studies investigating the link between vowel lenition and coarticulation (e.g. [12] for Japanese; [11] for English; [13] for Korean), it is hypothesised that traces of the lenited vowel remain in the acoustic signal via coarticulation with the adjacent consonant. Therefore, consonants that are rendered word-final due to vowel lenition are predicted to differ from canonical word-final consonants in terms of various acoustic parameters.
- Finally, on the basis of research carried out on the nature of vowel lenition, outlined earlier in this section, it is assumed that vowel lenition in CG is a phonetic process. This claim will be corroborated if vowel realisations in CG can be shown to fall at various points on a continuum and if vowel-to-consonant coarticulation is shown to take place in the language.

## Methods

### Materials

The material for the experiment consisted of 8 words or phrases embedded in carrier-sentences (see Table S1). They were produced 6 times by 8 speakers, resulting in a corpus of 384 sentences. Of the original 8 sentences, there were 7 which contained either /u/ or /i/ utterance-finally and preceded by /t/–as voiceless consonants have been shown to favour lenition [5], [44]. The decision to place the sequences utterance-finally was based on the assumption that vowels are more likely to be lenited if they are found at the end of an utterance [4] or adjacent to prosodic boundaries [15]. The sequences were constructed on the basis of the structure of two of the weak personal pronouns of Greek, /su/ (your) and /tu/ (his), as this experiment formed part of a larger study investigating CG enclitics. Single lexemes containing this sequence of sounds with stress on the same position were also included. For the /u/ sentences, stress was varied from the preantepenultimate syllable, to the antepenultimate and to the penultimate. For the /i/ sentences, stress was only varied from the antepenultimate to the penultimate syllable, as /i/ does not occur word-finally in enclitics.

Hypothesis (2ii) stated that possible traces of the lenited vowel might remain in the consonant, thus changing the acoustic properties of the–now word-final–consonant. Such changes would show that this consonant differs from canonical word-final consonants. Thus, a token with a canonical word-final /t/–[bit] (*bit*) was used as the control. As /s/ and /n/ are the only word-final consonants in CG [42], loanwords were used to investigate the properties of /t/. A monosyllable was selected in order to ensure that the vowel preceding the consonants in question would be unlikely to undergo lenition as it would necessarily carry stress. This token is considered to be a canonical manifestation of /t/ because it is utterance-final and is, thus, unlikely to be affected by following sounds. The term ‘canonical /t/’ refers to an alveolar consonant during the production of which there is a clear silence portion and a clear release portion manifested via a burst and aspiration. The set of sentences was randomised six times and the five most natural productions were selected by the experimenter for analysis, resulting in a corpus of forty productions per speaker (8 sequences * 5 productions).

### Procedure

#### Participants

Recordings were made of eight native speakers of CG, five male (XS, PK, SV, YP and LT) and three female (DD, AV and LL), aged 21–26. All speakers were monolingual and spoke the standard variety of CG (‘monolingual’ is used here in its minimal sense; this means that participants were brought up with CG as their only native language). The speakers were all students at the University of Cambridge at the time of the recording and had been living in the UK for at least 3 months prior to the experiment. None of the speakers reported any hearing or speech defects, and none had any previous training in phonetics, hence they were all naive to the purposes of the experiment. Verbal informed consent was obtained from all participants after the procedure was explained to them, and they were all reimbursed for their time. Ethics approval was not required for this study as it involved a non-invasive technique and the recordings are not publicly available.

#### Presentation of material

CG is not a written language and, due to lack of orthographic conventions, it was difficult to present the material to the participants in written form, because of fear that they might lapse into SG or speak unnaturally (see note 2 in Supporting Information File S1). It was also not possible to use the technique of ‘Say X again’ because the experimenter did not want to produce the sequences for the speakers to repeat. For want of a better method, the sentences were presented in written form using SG orthography, though following some conventions devised for CG, but speakers were not permitted to read them straight away. Instead, they were asked to read each sentence silently and then to look away from the paper. The experimenter would then ask a question to which they had to respond with the token in question. The question was: ‘What did you want to tell me?’. The assumption was that this technique would minimise any possible effects of the non-standard orthography of the sentence, as it required speakers to process the sentence before producing it. The method was successful in that in most cases speakers were able to answer the experimenter's questions without having to look at the paper again. Speakers were asked to repeat a sentence whenever the experimenter felt that they had not produced it with a neutral intonational pattern, and to speak at a normal pace.

#### Recordings

The recordings took place in the sound-treated room of the Phonetics Laboratory of Cambridge University in two-hour sessions per subject. Each speaker was recorded on a separate DAT tape into a Sony recorder. The material was then digitised at a sampling rate of 16 kHz onto a Silicon Graphics Unix workstation running the *Xwaves+* speech analysis package. The data were then converted to wav form so as to be analysed using PRAAT.

### Measurements

The first step in the analysis involved a visual inspection of the speech signal using PRAAT; this included the observation of waveforms and spectrograms, with the dynamic range settings held constant. The aim was to identify, with respect to specific acoustic criteria, whether the vowel in the sentence under investigation was present in the acoustic signal. The second step involved measuring various acoustic properties of the consonant. In order to provide an overview, the procedures followed are summarised below:

#### Vowels

The speech signal was examined visually and the presence of vowels was established by whether a periodic waveform corresponded to formant structure and vertical voicing striations in the spectrogram, together with an F0 trace. Each sound file was inspected separately and not all of these properties of the speech signal had to be manifested for the experimenter to determine that a vocalic sound was present. Aural examination of the signal was also performed by the experimenter, but results are not based on it, as native listeners tend to hear vowels which are not visually present in the signal. The classification of the various vowel sounds is explained in detail in the Results section.

#### Stop V_1_F2 frequency

**Figure 1:**
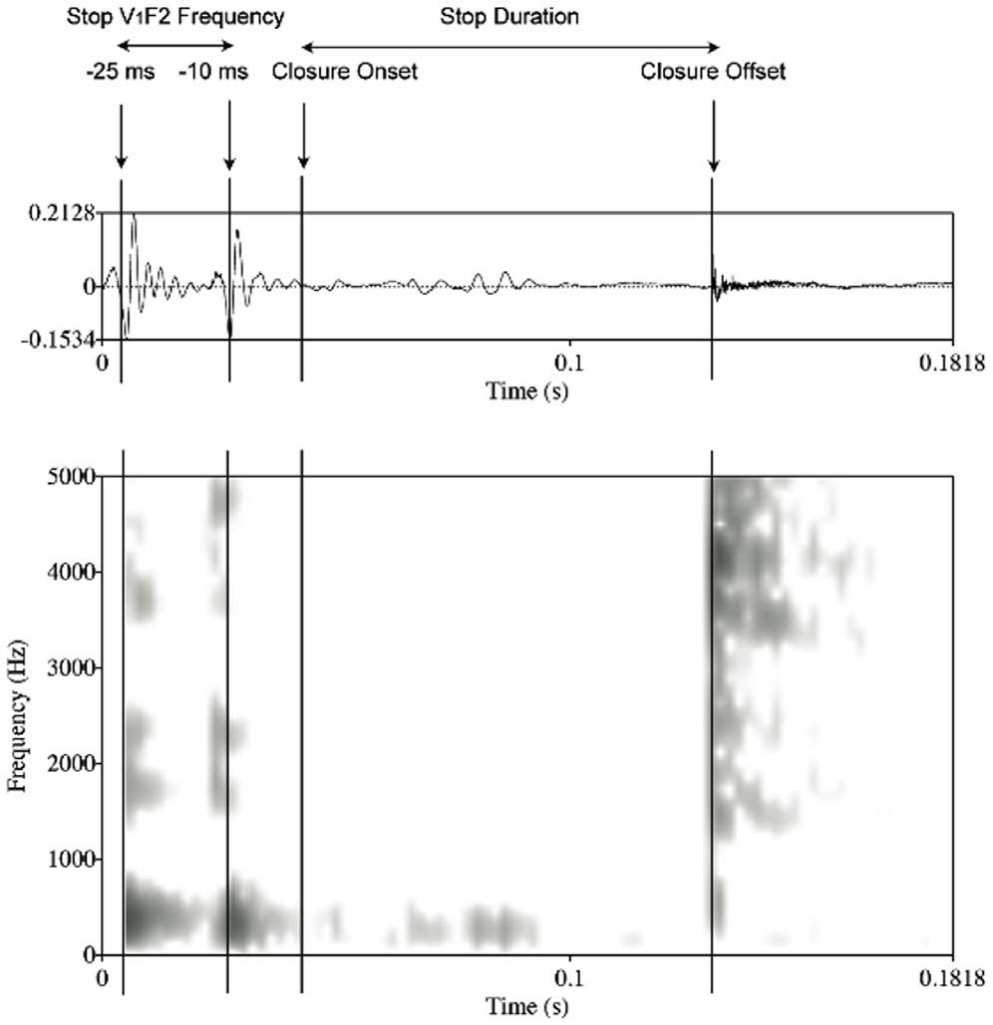
Waveform and spectrogram depicting how stop V_1_F2 and stop durational measurements were made. Vertical markers show the time window where the measurement was made.

V_1_F2 frequency was measured manually. LPC spectra were extracted from an interval of 25 ms at the offset of the preceding vowel. The marker was placed 10 ms prior to the end of periodicity in the waveform–corresponding to formant structure in the spectrogram–and PRAAT was used to add 25 ms towards the midpoint (see Figure 1 for an example). Reference was made to the spectrograms to ensure the validity of the spectral measurements.

#### Stop duration

**Figure 2:**
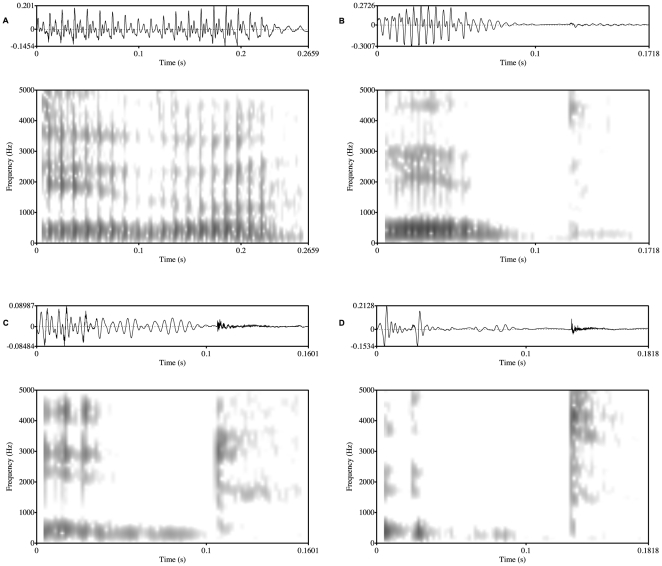
Waveforms and spectrograms of the sequence /itu/ depicting the various realisations of /u/. A) Full vowel, B) F1 vowel, C) Fricated vowel, and D) Elided vowel.

Stop duration was measured in milliseconds from the last periodic pulse preceding silence in the waveform, in conjunction with the disappearance of formant structure in the spectrogram, to the burst. In cases where the stop was produced with voicing or as an approximant, the main determinants for the calculation of duration were the disappearance of formant-structure, in the first case, and the drop in amplitude. VOT was not included in the durational measurements because not all stops were produced canonically, and, thus, did not exhibit a burst or aspiration (see Figure 2).

### Statistical analyses

Statistical analyses were carried out in SPSS using one-way repeated-measures ANOVAs.

#### Stop V_1_F2 frequency

Frequency data measured in Hz were normalised to account for anatomical differences between male and female speakers, and for within-speaker variability [45]. The procedure followed was a z-score transformation as detailed in Lobanov [46]. Firstly, the individual data points for a particular speaker's productions per condition were averaged in order to get the mean for the particular condition. The standard deviation for this mean was then computed. Finally, the overall mean was subtracted from the value of the individual token to be normalised, and the new value was divided by the standard deviation, thus yielding the normalised value. The Lobanov method was selected on the basis of the comparison carried out by Adank *et al*. [47] of various normalisation procedures used in language research. They found that the Lobanov method performed best at reducing anatomical/physiological variation and preserving phonemic variation.

As it was not possible to control for how speakers produced the material, very few instances of elided vowels were produced, resulting in a very small set on which it was not possible to run statistical tests. For this reason, instances of fricated vowels were also used in the analysis. The set of data includes productions from the 6 speakers who elided their vowels (DD, LL, AV, XS, YP and LT).

For this analysis, V_1_F2 offset acted as the dependent variable and the independent variable was the sound (/u/ or /i/), or lack thereof (in the case of word-final consonants), following the consonant. The ANOVA involved planned comparisons in which the two experimental groups, that is the set of data for which the stop occurs in the context of /u/ and the set of data for which it occurs in the context of /i/, were compared against the control group, that is the set of data in which the /t/ occurs word-finally. After this first comparison, the experimental groups were compared against each other. Thus, the independent variable had three levels: /u/, /i/ and canonical consonant.

#### Stop duration

As previously mentioned, not all types of stop realisation were produced by every speaker. Since it was not possible to merely delete speakers, as this would create a very small data set on which to run statistical tests, the missing data from these speakers were replaced by the mean of the other speakers' productions. This test included data from the same 6 speakers whose productions were used for the V_1_F2 onset test. For this analysis, consonant duration was the dependent variable, and the way in which the consonant was realised was the independent variable.

## Results

### Vowel Realisation Continuum

**Figure 3:**
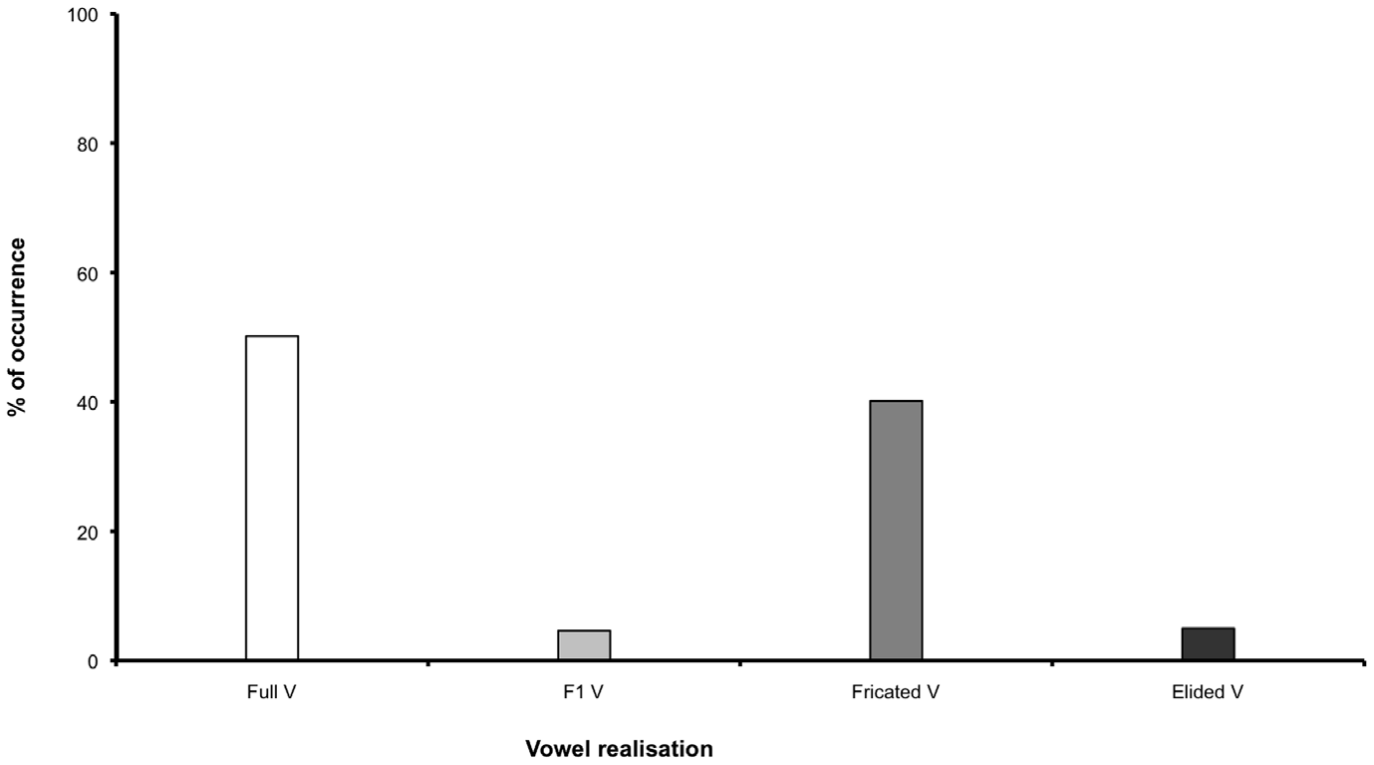
Bar graph depicting the percentage of occurrence of each vowel realisation.

Hypothesis (2i) stated that vowel lenition in CG is prevalent and hypothesis (2iii) claimed that it is gradient. In order to test these claims a visual inspection of the acoustic signal was carried out so as to ascertain whether vowels go through various stages before reaching elision. The hypotheses were corroborated by the results as lenition occurs in 48.92% of the data (see Table 1). Different realisations of vowels were obtained which were classified into four categories according to their common attributes (see Figures 2 and 3). It should be clarified that the classification of the realisations is not intended to mean that they do not form part of a continuum; rather, it is used as a way to handle complex data and as a tool to facilitate further analysis. On this note, it should be mentioned that, in some cases, a token could fall into more than one category, but it was classified according to where the majority of its features lay. The categories were:

**Table 1:** Cumulative, and per vowel, percentage of occurrence of vowel realisation.

Vowel realisation/Vowel context	/u/+/i/	/u/	/i/
Full vowel	50.7	49.0	55.0
F1 vowel	4.6	6.0	1.3
Fricated vowel	39.6	40.0	38.8
Elided vowel	5.0	5.0	5.0

#### (a) Full vowels:

Such vowels were produced with clear voicing and formant-structure, and they occured in 50.7% of the data (see Figure 2A).

#### (b) F1 vowels:

In such cases, occurring in 4.6% of the data, voicing was present in the waveform and an F0 contour could be traced in the acoustic record, but only the first formant was visible and audible in the spectrogram (see Figure 2B).

#### (c) Fricated vowels:

Fricated vowels were produced with no voicing but with their F2 and F3 following the aspiration of the preceding consonant. This realisation occured in 39.6% of the data (see Figure 2C).

#### (d) Elided vowels:

This category included instances of tokens where there was no visual evidence of a vowel in the acoustic record. Once again the dynamic range settings were held constant when inspecting the signal and this category includes instances of tokens where the formant transitions into an underlying vowel were not as pronounced as, and are much shorter than, the case of fricated vowels. Instances of this realisation were found in 5% of the data (see Figure 2D).

### Stop Duration

**Figure 4:**
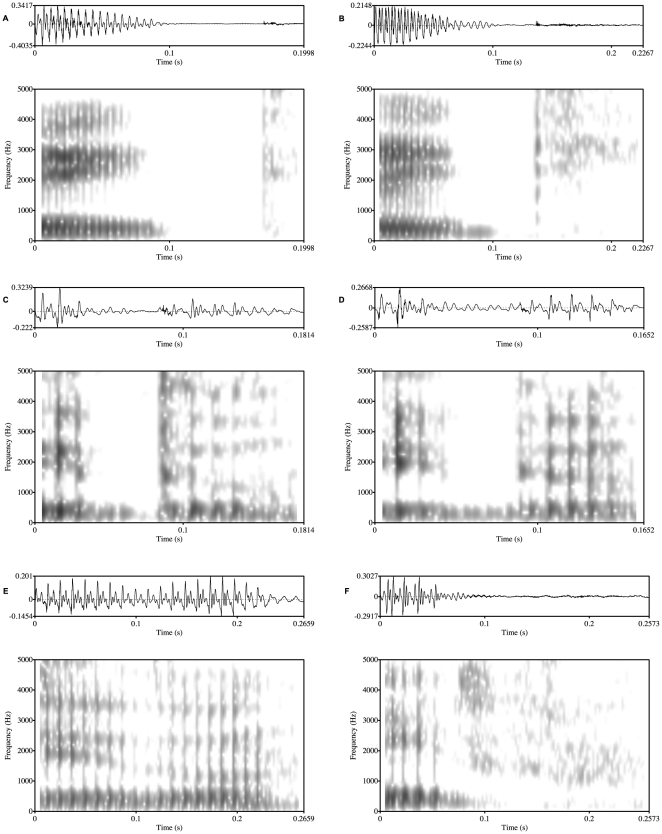
Waveforms and spectrograms of the sequence /itu/ depicting the various realisations of /t/. A) Canonical productions, B) Partially Voiced productions, C) Voiced-to-Burst productions, D) Voiced productions, E) Approximant productions, and F) Fricated productions.

**Figure 5:**
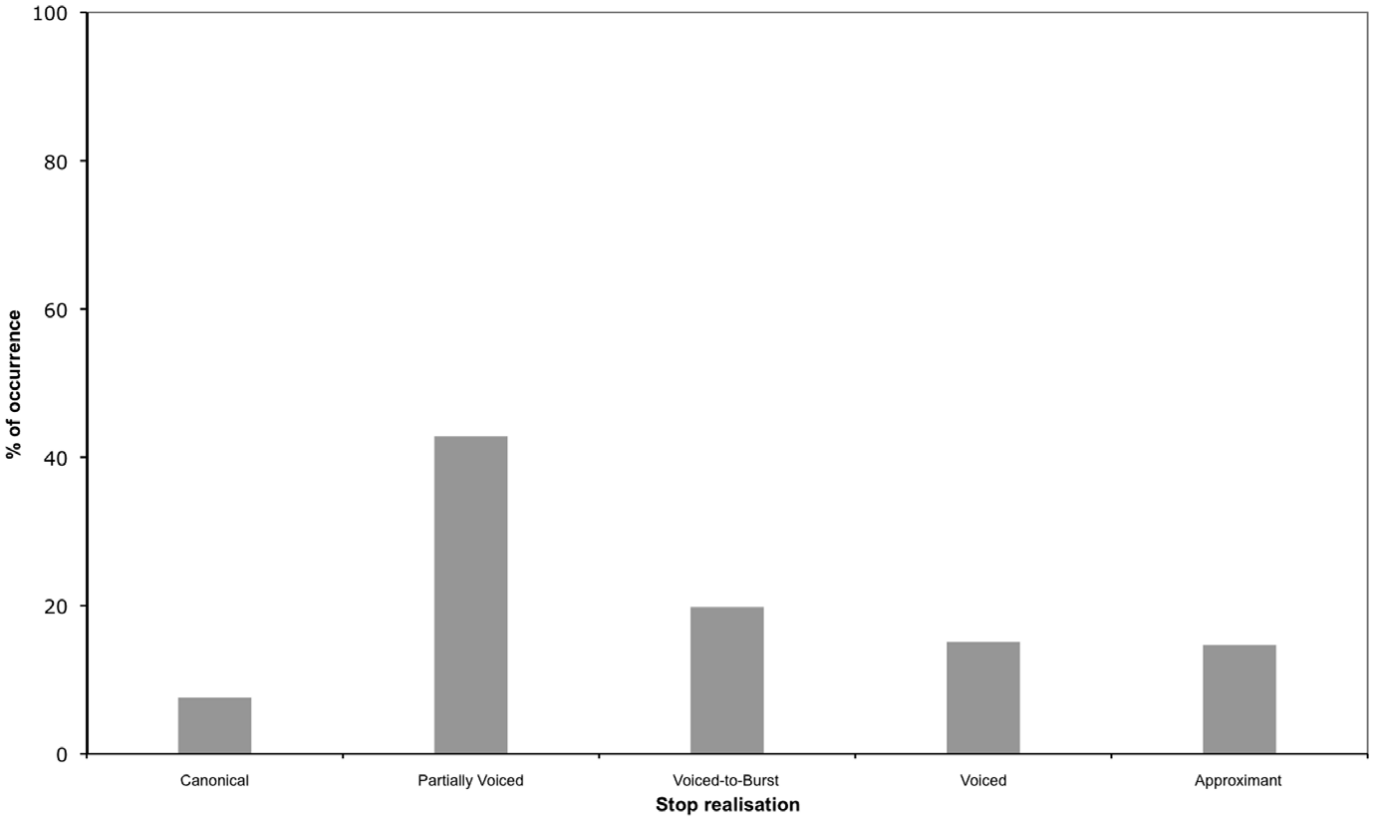
Bar graph depicting the percentage of occurrence of each stop realisation.

After visually inspecting the waveforms and spectrograms of the tokens, it became clear that the realisation of stops, like that of vowels, is variable in CG, as in other languages (e.g. [48] for English; [6] for Greek), thus offering experimental evidence for past impressionistic observations of the lenition of CG singletons [49] and corroborating previous studies on CG stops involving spontaneous speech [50]. /t/-realisations varied from approximant productions to canonical productions. As with vowels, stop realisations were also grouped into categories (see Figures 4 and 5):

#### (a) Canonical productions:

In such cases, there was complete silence during the closure period followed by a transient and release characteristics. This realisation occured in 7.6% of the data (see Figure 4A).

#### (b) Partially voiced productions:

For such productions, there was periodicity during about half of the closure duration but silence for the rest of the closure until the burst (see note 3 in Supporting Information File S1). This was the most frequently occurring realisation found in 42.8% of the data (see Figure 4B).

#### (c) Voiced-to-Burst productions:

Such stops were produced with periodicity during the entire closure period and with a transient but it was not possible to see higher formants. Instances of this realisation were found in 19.8% of the data (see Figure 4C).

#### (d) Voiced productions:

The difference between this realisation and realisation (c) is that, although there was voicing during the entire closure period and no higher formants were visible, there was no release transient. This realisation was found in 15.1% of the data (see Figure 4D).

#### (e) Approximant productions:

Such segments were fully voiced and, at least, the first three formants were visible in the spectrogram. Instances of this realisation occured in 14.7% of the data (see Figure 4E).

#### (f) Fricated productions:

In such cases, the airflow is sufficiently unobstructed for air to escape but the constriction area is small enough to generate frication (see Figure 4F).

**Figure 6:**
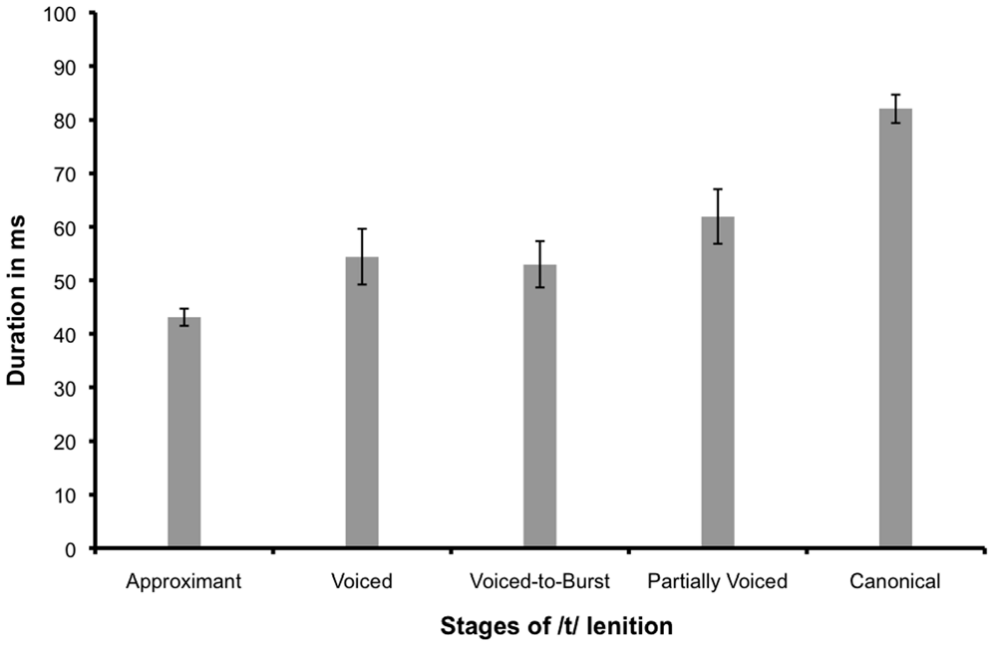
Bar graph depicting the duration in milliseconds of each type of /t/-realisation. Error bars indicate +/− one standard error.

Results also showed that duration was at its minimum when the stop was produced as an approximant (see note 4 in Supporting Information File S1)–about 43 ms–and at its maximum when produced canonically–about 82 ms (see Figure 6). This closure duration measurement included data from the six speakers who produced fricated and elided vowels. The instances of fricated stop were excluded from the analysis because they were deemed to be outliers (only 2 out of 280 stops were fricated). The analysis revealed that /t/-realisation has a highly significant effect on its duration (F(4, 20) = 24.559; p<0.001). Post-hoc Bonferroni tests revealed a robust distinction between the duration of a canonical stop and all other types of stop realisation (see Table 2 and note 5 in Supporting Information File S1), while the other types of stop realisation did not differ significantly from each other.

**Table 2:** P-values reflecting significant differences in the duration in ms of the various /t/-realisations.

/t/-realisation	Approximant	Voiced	Voiced-to-Burst	Partially Voiced	Canonical
Approximant	---------------				
Voiced	0.720	---------------			
Voiced-to-Burst	0.903	1.000	---------------		
Partially Voiced	0.104	1.000	0.496	---------------	
Canonical	0.001	0.032	0.007	0.059	---------------

**Figure 7:**
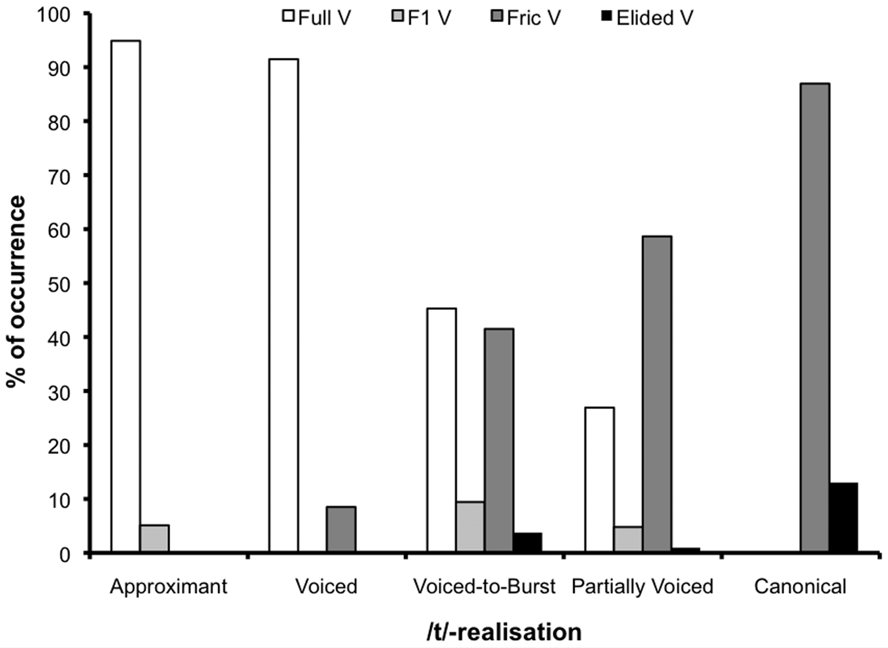
Bar graph depicting the percentage of occurrence of each vowel realisation per stop realisation.

In addition, there seems to be a relationship between stop realisation and vowel realisation (see Figure 7). Full vowels were present more often when the consonant was lenited, and elided more often when the consonant was produced canonically (see Figure 2A for an example). This point and its implications with respect to rhythm and aerodynamics are explored further in the Discussion.

### Stop V_1_F2 Offset

**Figure 8:**
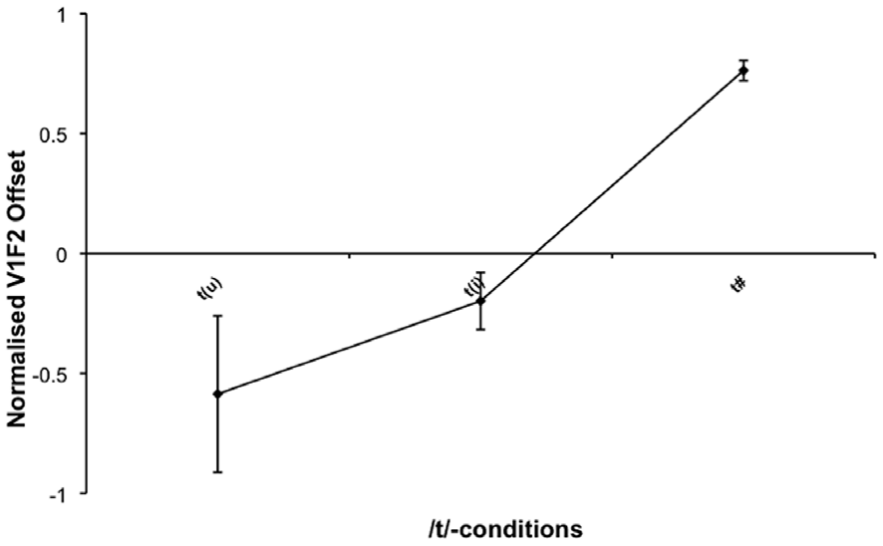
Line graph depicting the normalised V_1_F2 values for each of the /t/-conditions. Error bars indicate +/− one standard error.

V_2_ context in the case of /t/ had a highly significant effect on V_1_F2 Offset (F(2, 10) = 13.670; p = 0.001), indicating the existence of anticipatory vowel coarticulation (see Table 3 and Figure 8). The original hypothesis motivating the measurement of this particular spectral property was that the vowel that follows the consonant has an effect on the F2 offset of the vowel preceding the consonant. Planned comparisons were carried out between the three different contexts, which show that there exist robust distinctions between /t(u)/ and /t#/, as well as between /t(i)/ and /t#/ (see Table 4). Interestingly, the difference between /t(u)/ and /t(i)/ was not statistically significant.

**Table 3:** Mean frequencies in Hz and Standard Deviations for V_1_F2 in all /t/-conditions.

/t/-conditions	Mean Frequency	S.D.
/t(u)/	1947.4	232.7
/t(i)/	2057.9	288.8
/t#/	2184.6	291.4

**Table 4:** P-values reflecting significant differences between the V_1_F2 of /t/-conditions.

/t/-conditions	/t(u)/	/t(i)/	/t#/
/t(u)/	-------		
/t(i)/	0.236	-------	
/t#/	0.006	0.001	-------

## Discussion

### Vowel lenition in CG

Vowel lenition exists in many of the world's languages, including SG [2], [4], [5], [51], but considered to be relatively rare in CG [42]. The present study is the first to test the claim empirically and to offer significant insight regarding the frequency of vowel lenition in CG. The study shows that vowel lenition is common in CG, occurring in about 49.28% of the data (see note 6 in Supporting Information File S1), and provides evidence for the realisation of vowels in four different ways. However, one caveat is that although productions form four different clusters, the classification of the data is nevertheless arbitrary, as it is based on qualitative criteria. Moreover, as mentioned in the Results section, there are productions whose attributes fall into more than one category, but were still classified into a category according to where the majority of their features lay. For these reasons, the classification attempted in the aforementioned section should be viewed as a tool aimed to facilitate answering the research questions the present paper is concerned with regarding the nature of vowel lenition and its link with coarticulation. These questions are addressed in detail further on.

### Coarticulation

Previous research carried out on anticipatory coarticulation has shown that coarticulation with upcoming segments alters the acoustic properties of target segments (e.g. [27], [29], [30] for fricatives; e.g. [24], [28] for stops), even in cases when the vowel appears to be elided [12], [13]. Based on the fact that these acoustic alterations are perceptually salient (e.g. [12], [28], [36]), it was predicted in the Introduction that lenited vowels that have reached the stage of elision on the vowel realisation continuum would, nevertheless, coarticulate with their preceding consonant resulting in acoustic differences between a consonant rendered word-final because of vowel elision and a canonical word-final consonant. The prediction is corroborated by the results of the experiment described in the present paper.

The V_1_F2 Offset measurement showed that both /u/ and /i/ leave coarticulatory traces on the preceding stop to distinguish CV sequences from canonical word-final stops. This result is not in agreement with previous perceptual studies (e.g.[36], [37]), which found high rates of identification of /i/ in the context of /t/ or /d/ but low rates of /u/ identification, suggesting that a greater amount of coarticulation occurs between /i/ and /t/. However, the present study is in agreement with the results of Blumstein and Stevens [34] and Sereno *et al*. [28] whose subjects were able to correctly identify both /i/ and /u/ from stop bursts. This discrepancy might be due to the fact that no transition information was contained in the burst stimuli of the Bonneau [36] and Winitz *et al*. [35] studies. /t/ is a dental sound and, as a result, its burst will exhibit high-frequency spectral peaks. Perhaps listeners are predisposed to restoring an elided /i/ in such contexts barring information to the contrary. As Winitz *et al*. [35] suggest, the high burst for /t/ may be interpreted by the listener as /i/, or as Bonneau [36] puts it, the acute spectrum of /t/ might evoke an /i/ timbre. It appears that coarticulatory information in the transitions is very important for the identification of /u/. Bonneau [36] argued that the anticipation of lip rounding is not sufficient to lower the burst so as to allow identification of /u/ from /t/, suggesting that the tongue body cannot assume its target position for /u/ immediately after the burst release. It is, therefore, possible that the apical and velar movements are in conflict, thus preventing a sharp movement towards a steady-state F2 [35].

While early anticipatory coarticulation, as reflected in V_1_F2 offset measurements, is present in the data, the analysis showed that /u/ and /i/ are not significantly different from each other. Although there might be enough information in the V_1_F2 offset to indicate the possible underlying presence of a vowel, there might not be enough information to determine the identity of the vowel. In other words, this result might be a function of the fact that the measurement was taken too early in the acoustic record to determine the identity of the underlying vowel. This would mean that coarticulated /t/ is different from canonical /t/, but the two types of coarticulated /t/, i.e. /t(u)/ and /t(i)/, are not significantly different from each other.

The acoustic analysis of the /t/ data reveals similarities and differences in the coarticulatory behaviour of the consonant in CG and Korean. Mo [13] found that /t/ lenites 36% of the vowels. This result agrees with the results of the present study, which found very few instances of elided vowels in this context. The difference between Mo's study and the present study lies in the fact that, unlike what happens in Korean, voiced /t/ is never followed by a lenited vowel in the present study. An explanation for this result is given later in this section.

### Consonant lenition

The study described in this paper found that stop production is quite variable, with realisations ranging from fully lenited stops, resulting in approximants, to stops produced with a complete closure and a detectable burst. Also, the experiment has shown that consonant duration is at its minimum in the lenited cases and at its maximum when the target is achieved perfectly (see Results section). While canonical stop duration was shown to differ significantly from the durations of the other four stop realisations, these realisations were not shown to be significantly different from each other. Since the closure duration for the canonical consonant averages at 82 ms, it is reasonable to assume that the canonical form requires extra time to produce the necessary supraglottal pressure for a release burst.

However, the intraoral pressure needs about 20 ms to increase to within 2 cm H_2_O, after which point glottal airflow decreases rapidly [52]. Moreover, if the closure extends beyond 50 ms, the intraoral pressure reaches a value equal to the subglottal pressure, with the glottal airflow decreasing to zero. With this in mind, it cannot be argued that the canonical form differs from the other four in that it is the only one to have enough time to produce the necessary supraglottal pressure for a release burst. It appears, then, that while there is a continuum of realisations, they are not necessarily based on duration, especially given the fact that the duration of a voiced stop involving a burst lies between that of a voiced stop without a burst and that of a partially voiced stop. If that were the case, in the case of the approximant it would be that the duration is too short, not giving the articulation enough time for an increase in oral pressure sufficient to stop vocal fold vibration. In the case of the voiced stop, a slightly longer duration results in a reduction in amplitude with the higher formants disappearing completely. In the case of the released voiced stop, the longer duration would allow pressure build-up for a burst but not for vocal-fold abduction. In the case of the partially voiced stop, even longer duration results in the cessation of voicing. Finally, the canonical stop is long enough for an increase in oral pressure sufficient to prevent voicing and result in a burst. Whether, in fact, this continuum is based on duration and whether there are differences in airflow and subglottal and supraglottal pressure cannot be determined on the basis of this acoustic experiment, but could be addressed in future research using airflow and pressure measurements. While the hypothesis cannot be discarded, it is possible that the different consonant realisations are due to a combination of changes in the supralaryngeal and laryngeal activities. This final possibility is explored in further detail later on in this section.

### The Nature of Vowel Lenition in CG

Stemming from the question of whether vowel lenition in CG is gradient or discrete – formulated in hypothesis (2iii) – one motivation for the present study was to gain insight into this process and into its implications for phonetics and phonology. Following on from this, vowel lenition in CG was predicted to be a phonetic process, resulting in gradient outputs, similar to what has been found for comparable processes in other languages.

As shown in the Results, and mentioned in passing in this Discussion, vowel lenition is common in CG, with vowels realised in, at least, four different ways. However, despite the fact that the process is very prevalent, and a realisation continuum appears to emerge that is similar to other linguistic systems such as Japanese [21] or SG [4], [5], the results do not clearly argue for gradience when examined on the surface. The continuum is not scalar and productions form four different clusters. The fact that productions form clusters could also indicate discreteness, as long as each cluster of productions was completely different from the others. However, as emphasised previously, the analysis made it essential for certain borderline productions to be classified in a category according to the majority of their features; this situation suggests that arguing for a categorical choice between four possible realisations is not plausible, as some productions did not fall unambiguously under a particular category. Therefore, unless as many categories are postulated as there are productions, the process would be best treated as gradient.

As demonstrated by other studies – reviewed in the Introduction – which investigated the link between vowel lenition and coarticulation, changes in the spectral or temporal properties of consonants caused by adjacent vowels undergoing lenition indicate that the vowel is not deleted but, rather, is still present in the acoustic record and, therefore, in the articulatory event. The results of the present study also offer support for the phonetic account as strong evidence for vowel-to-consonant coarticulation, even in extreme cases of apparent vowel elision, has emerged from the analyses of the data. Coupled with the coarticulation results, the gradient outputs indicate that the process can be accounted for within the AP framework. According to this theory, the gestures for the vowel and consonant overlap in time with the resulting production manifesting properties of both to varying degrees depending on the extent of overlap. The process is the result of the hiding of the vowel's oral and glottal gestures by those of the preceding consonant's, thus resembling what happens in Japanese and Korean (e.g. [13], [14]).

Another angle to be considered in the discussion of the phonetic nature of the process is that it might facilitate perception. Firstly, the existence of vowel-to-consonant coarticulation might aid vowel identification. Experiments have shown that spectral changes caused by overlapping gestures are sufficient cues for the identification of the vowel, thus establishing a link between production and perception (see Introduction). This vowel-to-consonant coarticulation appears to have perceptual salience in CG as well based on native-listener intuition. Moreover, it is possible that it is important for listeners to be in contact with the various stages of the vowel realisation continuum in order to become accustomed to the acoustic cues left in the preceding consonants; at each stage of the continuum native listeners might be aided by such coarticulatory strategies so as to perceive the missing vowel correctly. Of course, this interpretation implies that at some point the process might become phonological, as listeners will be more adept at picking out the appropriate perceptual cues even in cases of extreme vowel lenition, without needing to have contact with intermediate stages. It should be emphasised that the possibility of the process becoming phonological is not limited to there being only two vowel ‘realisations’: vowel presence or vowel absence. It is also possible for the process to be phonological as long as there are discrete categories of vowel realisation.

### Routes to lenition

One of the main outcomes of the present study is that there are continua of realisations for both vowels and consonants. Moreover, there appears to be a relationship between consonant realisation and vowel realisation, with vowels preserved more often when the consonant is lenited, and elided more often when the target for the consonant is fully achieved. Thus, this relationship is not a simple one, since it is not that the speaker is not being careful when speaking in the case of vowels but careful in the case of consonants. There seems to be a complex interaction between the articulations of the two segments.

Coming back now to the matter of the interaction between consonant and vowel articulation, it appears that there are two routes to lenition in CG, with vowel realisation dependent on consonant realisation. If there is a complete closure for a stop, voicing ceases and the vowel is voiceless; if the closure is not achieved, voicing is preserved and the vowel is voiced. As obstruents involve an accumulation of air in the oral cavity, which causes a decrease in the transglottal pressure drop and, consequently a decrease in airflow through the glottis, they inhibit voicing [53]. Moreover, close vowels give rise to longer VOT [54] because the oral pressure from the stop decays more slowly due to a greater resistance to airflow caused by their narrow constriction [55]. The situation in the present data is accounted for if the account proposed by Ohala [53] is taken one step further to include instances when the voicing does not resume because the short vocalic gesture has been masked by the consonantal gesture.

However, the two routes to lenition are not dependent on distinct laryngeal activities. If a laryngeal gesture is not strong enough to either preserve or prevent voicing, a fully achieved lingual gesture will result in complete closure, which will then result in a lenited – devoiced – vowel. In other words, assuming the default glottal configuration for speaking is adduction of the vocal folds for voicing, with a voiceless sound needing a temporary abduction gesture, then a less than complete abduction gesture which is ‘marginal’ with respect to voicing, and a complete oral closure, will result in the reduction in transglottal flow. This reduction will result in the cessation of voicing and will have as a consequence the lenition of the following vowel – also assuming the lack of an energetic adduction gesture to reverse the marginal setting. If, on the other hand, the weak laryngeal gesture is accompanied by a weak lingual gesture, the oral target for the consonant will not be fully achieved, resulting in a lenited consonant, which will, in turn, be followed by a full vowel. In other words, if, given the same marginal abduction of the vocal folds, the tongue does not reach its target, which is the alveolar ridge in this case, a lenited stop will be produced. Following on from this, since the vocal folds are still vibrating, a full – voiced – vowel will ensue. Thus, the phenomenon can ve viewed as one where the supralaryngeal articulation gives rise to the aerodynamic conditions which result in passive devoicing.

### Conclusions

In sum, this paper has presented quantitative data regarding the nature of vowel lenition in CG and the link between vowel lenition and coarticulation in CG. Vowel lenition was hypothesised to involve gradient variation from canonical vowels to lenited vowels and this hypothesis was confirmed as various different vocalic realisations were obtained. The great degree of anticipatory vowel-to-consonant coarticulation also supports this hypothesis, as it demonstrates that the vowel is still present, in some form, in the acoustic record. There is scope for future work in terms of lenition in non-close vowels. Such investigation might reveal that lenition is even more widespread than originally assumed. Lenition in close vowels is almost expected given their intrinsic characteristics [9], [19], [20]. Non-close vowels, however, do not exhibit such characteristics and an investigation of whether they are lenited would be worth pursuing. If they are in fact lenited, the cause of the lenition process might not lie solely in the blending of gestures and other aerodynamic effects–although these might also hold and be responsible for lenition–but, rather, in a process of change in the linguistic system which is moving towards final vowel lenition across the board. There is also scope for investigating the phenomena perceptually in order to determine whether the acoustic vocalic traces are perceptually salient.

The study has also revealed the existence of a continuum of consonant realisations by showing that CG stops can be realised with variability in both laryngeal and lingual activity. What is more, there is a relation between the type of consonantal realisation and the duration of the consonant, with canonical consonants being the longest and fully lenited consonants being the shortest. It is difficult to maintain vocal fold vibration during obstruent production and, as a consequence, voiced obstruents tend to be shorter. Future studies could investigate whether the continuum of stop realisations revealed in the study is one based on duration. In the Discussion, it was hypothesised that perhaps longer duration is essential for creating the necessary supraglottal pressure which would lead to a release burst. While all types of stop realisation were longer than the minimum time required to produce the necessary intraoral pressure for a release burst, it cannot be argued with certainty that all stop realisations had similar airflow and subglottal pressure values without airflow and pressure measurements.

A limitation of this study is that the analysis conducted on vowel realisation had to be based on qualitative measures. While it has been specified throughout that this was merely a tool used to carry out further acoustic analysis, it would be worth pursuing the development of methods to quantify vowel lenition. Temporal measures are not appropriate given the fact that the vowel is elided in the last stage of the continuum, and spectral measures are not appropriate both for *elided* vowels but also for *F1* and *fricated vowels*, since *F1 vowels* exhibit only the first formant and *fricated vowels* exhibit only F2 and F3. Perhaps an index could be devised for a much bigger sample than the data set collected in the present study which should be tested for its correspondence to the realisations.

The main finding of the study is that there are two routes to lenition in CG; one route involves lenition of the consonant with canonical realisation of the vowel, while the other involves canonical realisation of the consonant coupled with lenition of the vowel. The experiment showed that canonical consonants and full vowels never co-occur–the same holds for fully lenited consonants and vowels. Hence, the voicelessness exhibited in the sequences under investigation was explained as an instance of passive devoicing as a consequence of the supralaryngeal articulation and a marginal glottal setting.

## Supporting Information

File S1 Endnotes.
(0.03 MB DOC)

Table S1 Test sequences and their glosses for each of the Vowel, Stress, and Clitic conditions.
(63 KB PDF)
