# Supplementary material for: Routes to Lenition: An Acoustic Study
Source: PLoS One. 2010 Mar 23;5(3):e9828. doi: 10.1371/journal.pone.0009828 (PMC2843704; doi:10.1371/journal.pone.0009828)
Supplement: File S1 — Endnotes. (0.03 MB DOC) [file pone.0009828.s001.doc]

1. Vowel reduction, in the sense of movement towards a less peripheral vowel space, i.e. towards more centralised vowels, is also present in Greek (e.g.[1,2]). More centralisation has been shown for /i/ than for /u/ (e.g. [3]). Nevertheless, the investigation of vowel centralisation is beyond the scope of the present study.

2. The official language of Cyprus is SG. This variety acts the medium for education and the media. However, the mother-tongue of Cypriots is CG, and is the medium for everyday communication.

3. The amount of periodicity varied from periodicity for about half of the closure duration to periodicity throughout most of the closure.

4. Measuring approximant duration was more difficult than measuring the closure period for a stop realisation. The criterion used for the onset of the approximant was a drop in amplitude coming out of the previous vowel and for the offset a rise in amplitude going into the next vowel.

5. The distinction between canonical stop and partially voiced stop approaches significance when running planned comparisons (p = 0.059) but is significant when running a one-tailed t-test (p = 0.002).

6. This number refers to realisations ranging from F1 vowels to elided vowels.

## References

1. Baltazani M (2007) Prosodic rhythm and the status of vowel reduction in Greek. Selected papers on theoretical and applied linguistics from 17th International Symposium on Theoretical and Applied Linguistics: 31-43.

2. Nicolaidis K (2003) Acoustic variability of vowels in Greek spontaneous speech. Proceedings of the 15th International Congress of the Phonetic Sciences: 3221-3224.

3. Fourakis M, Botinis A, Katsaiti M (1999) Acoustic characteristics of Greek vowels. Phonetica 56: 28-43.
