# Supplementary material for: Routes to Lenition: An Acoustic Study
Source: PLoS One. 2010 Mar 23;5(3):e9828. doi: 10.1371/journal.pone.0009828 (PMC2843704; doi:10.1371/journal.pone.0009828)
Supplement: Table S1 — Test sequences and their glosses for each of the Vowel, Stress, and Clitic conditions. (63 KB PDF) [file pone.0009828.s002.pdf]

Table 1: Test sequences and their glosses for each of the Vowel, Stress, and Clitic conditions.

/t/	u/		/i/
Stress condition	Clitic	Non-clitic controls	Non-clitic
Pre-antepenultimate	[ɛfː'ɐnisi tu]		
(PREANT)	*appearance its*		
Antepenultimate	[plɐstis'ini tu]	[x'ɐsitu]	[ɐp'oriti]
(ANT)	*play-dough its*	*person's surname*	*classified/secret*
Penultimate	[ɛfç'i tu]	[mɛsɐɾ'itu]	[mɛs'iti]
(PEN)	*wish his*	*person's surname*	*estate agent (gen)*
